# E-norms and AI in clinical neurophysiology

**DOI:** 10.1016/j.cnp.2024.12.001

**Published:** 2024-12-04

**Authors:** Joe F. Jabre

**Affiliations:** Department of Public Health and Community Medicine, Tufts University School of Medicine, Boston, MA, United States

**Keywords:** E-norms, AI, ChatGPT, EMG Nerve Conductions, Normal Values

## Abstract

**Objective:**

To describe the use of Artificial Intelligence (AI) to automate the e-norms method, a technique used to derive normative data from patient studies, mixed datasets that contain both normal and abnormal data. Multiple studies have shown that normal values collected with the e-norms method compare favorably with those collected from healthy volunteers using traditional methods.

**Methods:**

OpenAI’s ChatGPT was used by the author to build a Python script to automate the e-norms method’s plateau identification, the area of the e-norms curve where a variable’s normal values lie. To date, e-norms plateau identification has been done visually using an Excel Macro developed for that purpose.

**Results:**

E-norms normal values derived from the visual e-norms plateau identification with the Excel Macro compared favorably with those derived from the OpenAI’s Python script developed by the author to automate the e-norms plateau identification.

**Conclusions:**

OpenAI’s ChatGPT Python scripts can be developed by users with little to no experience in programming to automate the collection of e-norms normal values.

**Significance:**

A Neurologist with no experience in programming, and a limited knowledge of statistics was able to develop this on his own without the help of any programmers or statisticians.

## Highlights

• The author used AI to develop a Python script to automate the e-norms method to calculate normal values.
• He was able to do that with no experience in programming, and only a limited knowledge of statistics.
• With the tools it offers to healthcare providers, AI will revolutionize and democratize biomedical research.

## 1 Introduction

In this work I describe the use of Artificial Intelligence (AI) to automate a technique I developed to derive normative data from patient studies, mixed datasets that contain both normal and abnormal data, and refer to as the e-norms method(Jabre et al., 2015).

The method derives a variable’s normal values by plotting its sorted data as a line graph, the plot revealing an inverted S curve with an ascending slope in the beginning, a rather flat part or plateau in the middle where the slope is relatively constant, and an ascending slope at the end.

Multiple studies have shown that a variable’s data that lies within the plateau part of an e-norms curve where datapoints show the smallest first order difference between them, compares favorably with normal data for that same variable collected from healthy volunteers using traditional methods(Pitt and Jabre, 2017, Punga et al., 2019, Jabre and Bland, 2021, Shammas and Jabre, 2020, Nandedkar et al., 2015, Zaccarini et al., 2016).

The purpose of this work however was not to validate (or invalidate) the e-norms’ method and those that appeared following its first publication using a similar approach to collect normal values from patient studies (Nandedkar et al., 2018, Nandedkar et al., 2021, Reijntjes et al., 2021). A recent 2024 peer reviewed study by Dunker et al. (Dunker et al., 2024) has done just that by comparing e-norms to traditionally derived reference limits concluding that “with small adaptations, the e-norms method adequately replicates traditionally derived reference limits, and is a viable method to produce reference limits from historical datasets”. These works will not be discussed here as they lie outside the scope of this study.

Rather, in this work, the e-norms method was used because it proved fertile grounds to use OpenAI’s ChatGPT to compare the results obtained from an Excel Macro where the plateau of an e-norms curve is identified visually, to those obtained from a Python script developed by the author using OpenAI’s ChatGPT in an effort to standardize the plateau identification of an e-norms curve.

This work was also in no small part meant to show that a Neurologist with no experience in programming, and a limited knowledge of statistics, can develop this on his own without the help of any programmers or statisticians.

Visually identifying the e-norms plateau in the Excel Macro has to date been done with the help of a first order derivative of a variable’s data (i.e. variable 2-variable 1, variable 3-variable 2, variable 4-variable 3 etc..). An e-norms web app (eNorms, n.d.) developed by the author, can also be used for the same purpose, but was not used in the course of this work.

The Excel Macro plots this first order derivative using the area where it is lowest to identify the plateau part of the curve where the variable’s datapoints show the smallest consecutive difference between them, a property I refer to as the e-norms clustering behavior (Jabre, 2018).

Once the left and right inflection points that delineate the plateau are identified by the user, their X coordinates are inputted in the Macro, and the descriptive statistics of the variable’s datapoints that lie between them are calculated. The inflection points are areas where the e-norms curve transitions from a region where differences between consecutive datapoints are large, to another where these consecutive differences are small and vice versa, i.e. transitions from small to large comsecutive differences.

**Fig. 1:**
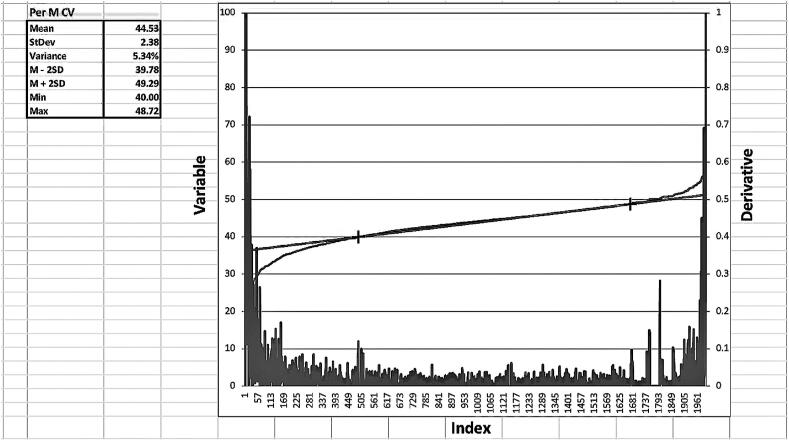
Plot of the sorted Peroneal motor conduction velocity (Per M CV) data revealing an inverted S curve. The inflections points at the beginning and end of the plateau are visually identified with the assistance of a low first order derivative below it. The X axis coordinates of the inflection points are then inputted into the Excel Macro that proceeds to generate the descriptive statistics of the variable under study seen in the left inset. (StDev is Standard Deviation).

Fig. 1 shows the Excel Macro and the descriptive statistics of the plateau data derived from an Excel file that contains 2000 Peroneal Motor Conduction Velocities (Per M CV) anonymized data without any protected health information (PHI) collected from clinical studies with both normal and abnormal findings, and approved by the local ethics committee.

While a 2020 study of the plateau’s visual identification by different users with the Excel Macro showed a good interrater reliability, as expected, there were subjective differences between the raters (Earle and Jabre, 2020).

The aim of this work is to describe the author’s own experience in the use of OpenAI’s GPT-4 to automate the identification of the e-norms plateau in an effort to avoid subjective differences one can encounter with visual plateau identification.

GPT-4 is an AI large language model (LLM) (Clusmann et al., 2023) that was trained on 175 billion words. OpenAI’s next generation GPT will reportedly have between 4.6 and 17 trillion words, but had not been released at the time of this writing. GPT stands for Generative Pre-trained Transformer, an advanced form of AI technology with the ability to generate human-like text in response to queries and inputs it receives.

## 2 Methods

The author, a Neurologist trained in clinical neurophysiology but with no experience in programming, became interested in AI from a fortuitous coming across Mustafa Suleyman’s “The Coming Wave” book (Damai, 2024) and set out to automate the e-norms plateau identification to replicate the workflow used in the e-norms Excel Macro to identify the e-norms plateau.

He first instructed GPT-4 to create a line graph of an uploaded Excel file that contains the Per M CV variable sorted ascendingly.

He then asked GPT-4 to identify and highlight all instances of the curve where the difference between consecutive datapoints is at its lowest to identify the flat or plateau part of it.

Following that he asked it to calculate the mean ± 1 SD of the datapoints where the difference between them was at its lowest given that the area between ± 1SD of a normally distributed curve contains 68 % of the normal values of a variable, that is, the plateau part of the curve.

Then he asked GPT-4 to use linear interpolation to get the X and Y coordinates of the first and last points where the ± 1 SD lines intersect with the curve to compare these values with the coordinates of the visually identified coordinates in the Excel Macro.

He then asked GPT-4 to calculate the mean and standard deviation of the data points that lie between the intersection of the ±1 SD lines with the curve to derive the e-norms normal values descriptive statistics of the variable under study and compare those to the ones derived from the Excel Macro.

While verifying the step-by-step interpretation with each input to GPT-4 to ensure its proper execution, the author asked GPT-4 to generate a Python script of these steps.

An important point to keep in mind is that GPT-4 was less likely to make errors on its response to inputs when those were given as step-by-step instructions, rather than grouped in a continuous set.

The following is a summary of the Python script generated by GPT-4 in response to the author’s inputs:

“**Imports Libraries**: It imports necessary libraries to the script like pandas for data manipulation, numpy for numerical operations, matplotlib.pyplot for plotting, and interp1d from scipy.interpolate for interpolation functions.

**Defines Main Function**: The program defines a main() function that encapsulates the primary workflow.

**Loads Data from an Excel File**: It reads data from a specified Excel file into a DataFrame using pandas. The file path is hardcoded and needs to be changed to match the user’s computer path to the file being studied.

**Dynamic Column Handling**: This dynamically identifies the first column of the data to perform operations, avoiding hardcoding the column name.

**Calculates Differences**: Used to calculate the differences between consecutive data points in the identified column and store these in a new column called 'Difference'. It also computes the absolute values of these differences to identify minimal changes.

**Identifies Minimal Changes**: Finds the minimum value in the 'Abs Difference' column and identifies all indices where this minimum difference occurs.

**Statistics on Minimum Differences**: For the points corresponding to minimal differences, it calculates the mean and standard deviation.

**Standard Deviation Calculations**: From the mean of the minimal differences, it computes values for one standard deviation above and below this mean.

**Filters Data**: Filters the dataset to include only those points that fall within one standard deviation above and below the mean of the minimal differences.

**Statistics**: For the filtered data points, it computes further statistics like mean, standard deviation, and +/- two standard deviations.

**Interpolation and Intersection Finding**: Performs linear interpolation on the entire data set to create a smooth curve. It then identifies points of intersection between this interpolated curve and the lines defined by one standard deviation above and below the mean of minimal differences.

**Plotting**: Plots the data, highlighting the filtered points, the standard deviation lines, and the intersections. Several details like mean, standard deviation, min, and max values of the filtered data are annotated on the plot.

**Execution Control**: The script checks if it is being run directly (not imported) and then calls the main() function.”.

**Fig. 2:**
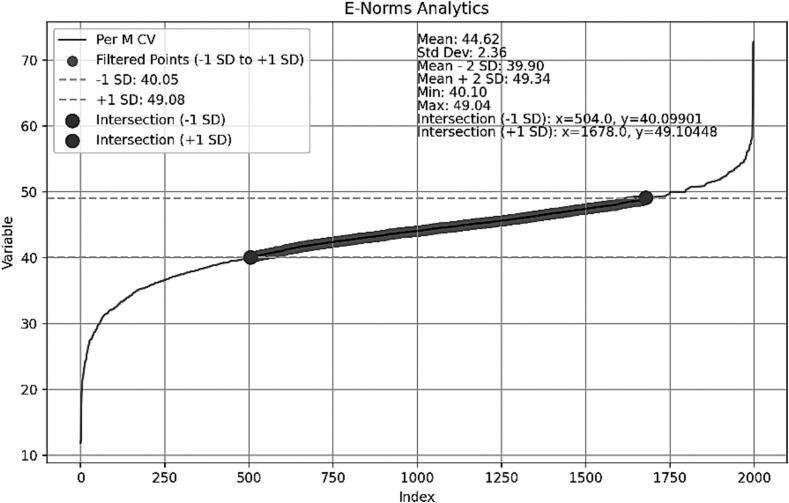
Plot obtained from the GPT-4 Python script analysis of the Per M CV data showing the inverted S curve with the filtered points delineated by the ± 1 SD hatched lines identifying the plateau part of the curve, and the table above it showing the descriptive statistics of the Per M CV normal values in the e-norms plateau.

The output of the GPT-4′s Python script from the Per M CV data is shown in Fig. 2.

Table 1 shows the comparison of the visual and GPT-4 automated script for the Per M CV data analyzed.

**Table 1:** Side by Side comparison of the descriptive statistics of the Per M CV data in the plateau part of the curve when the plateau was identified visually (Visual) and when identified using the GPT-4 AI script developed (GPT-4) columns.

Per M CV	Visual	GPT-4
Mean	44.53	44.62
SD	2.38	2.36
M − 2SD	39.78	39.90
M + 2SD	49.29	49.34
Min	40.00	40.10
Max	48.72	49.04

Once proof of principle of the visual Per M CV plateau comparison to the automated plateau generated by GPT-4′s Python script was obtained, the author proceeded to do a larger validation study by comparing 10 e-norms analysis of anonymized real data where the e-norms plateau was visually identified, to the same data analyzed with the plateau automatically identified with GPT-4′s Python script.

To analyze the data for significant differences between the outputs of both methods, the author asked GPT-4 to develop a separate Python script to perform this statistical analysis.

That script was developed as follows:

The author first extracted the visually identified plateau data of each of the 10 datasets he analyzed using the Excel Macro, here referred to as Method1, to compare those to the plateau data extracted from the GPT-4 Python script, here referred to as Method2, and built a single Excel file with these two methods side by side to analyze Method1 and Method2 for statistical difference.

Given that the number of values within the plateau obtained with each method (the Macro and the Python script) were not the same, and that some of the data were normally distributed and others were not, the following statistical methods were used to determine statistical significance:

*Independent t-test/Welsh’s t-test*: This test is suitable for independent, normally distributed data and is used to detect whether the means of the two groups are significantly different from each other.

*Mann-Whitney U Test*: This test is suitable for non-parametric data, or when normality is not assumed to find out if the ranks of the data differ between the two groups.

*Levene's Test*: This test is used to assess the equality of variances for a variable calculated for two or more groups. Given that the standard deviation is derived from variance, significant differences in variances imply differences in standard deviation.

The following is a summary of the Python script generated by GPT-4 for the comparison of Method1 and Method2 to determine statistical significance in response to the author’s inputs:

“**Import Libraries**: The script imports necessary libraries: pandas for data manipulation and scipy.stats for statistical tests.

**Read the Data**: The script reads an Excel file using pandas and stores it in a DataFrame (df). The file path is hardcoded in the script.

**Extract Data for Methods**: The script extracts the data for two methods (Method1 and Method2) from the DataFrame, dropping any NaN values.

**Calculate Descriptive Statistics**: It calculates the mean, standard deviation, minimum, and maximum for both methods.

**Print Descriptive Statistics**: The script prints the calculated descriptive statistics.

**Independent *t*-test**: It performs an independent *t*-test to compare the means of the two methods, assuming unequal variances.

**Mann-Whitney U Test**: The script uses the Mann-Whitney U Test to compare the distributions of the two methods.

**Levene's Test**: It performs Levene's Test to compare the variances of the two methods.

**Interpretation of Results**: The script interprets the results of the statistical tests based on a significance level (alpha = 0.05).

## 3 Results

Comparison of the results obtained from methods 1 and 2 can be seen in Table 2.

**Table 2:** Independent *t*-test, Mann-Whitney *U* test, and Levene’s test comparison for statistical significance of the Excel Macro and GPT-4 Python script derived e-norms. EP = Evoked Potentials, CV = Conduction Velocity, Sig Diff = Significant Difference, No Sig Diff = No Significant Difference.

	Independent *t*-test	Mann-Whitney U Test	Levene’s Test
**EP Latency**	**No Sig Diff**	**No Sig Diff**	**No Sig Diff**
Stats	t-stat: 0.0-P val 1.0	U Test: 3784.5-P val: 1.0	Stat: 0.0-P val: 1.0
**Sensory CV**	**No Sig Diff**	**No Sig Diff**	**No Sig Diff**
Stats	t-stat:0.73-P val: 0.47	U Test: 1053.5-P val: 0.60	Stat: 0.75-Pval: 0.39
**Motor Amplitude**	**No Sig Diff**	**No Sig Diff**	Sig Diff
Stats	t-stat: 1.45-P val: 0.15	U Test: 141198-P val: 0.08	Stat: 26.85-Pval: 2.63
**Sensory Amplitude**	**No Sig Diff**	**No Sig Diff**	**No Sig Diff**
Stats	t-stat: 0.83-P val 0.41	U Test: 83190.5-P val: 0.20	Stat: 1.42-P val: 0.23
**Eye Axial Length**	**No Sig Diff**	**No Sig Diff**	Sig Diff
Stats	t-stat: 1.75-P val: 0.08	U Test: 37957.5-P val: 0.14	Stat: 17.12-Pval: 4.08
**Motor Latency**	**No Sig Diff**	**No Sig Diff**	**No Sig Diff**
Stats	t-stat: 0.0-P val: 1.0	U Test: 76440.5-P val: 1.0	Stat: 0.0-Pval: 1.0
**EP Latency**	**No Sig Diff**	**No Sig Diff**	**No Sig Diff**
Stats	t-stat: 0.28-P val: 0.78	U Test: 7182-P val: 0.92	Stat: 2.74-Pval: 0.10
**Motor Amplitude**	**No Sig Diff**	**No Sig Diff**	Sig Diff
Stats	t-stat: 1.93-P val: 0.05	U Test: 70537.5-P val: 0.30	Stat: 132.42-Pval: 2.26
**Motor CV**	**No Sig Diff**	**No Sig Diff**	Sig Diff
Stats	t-stat: 0.19-P val: 0.86	U Test: 88126.5-P val: 0.59	Stat: 141.43-Pval: 2.44
**Motor Amplitude**	**No Sig Diff**	**No Sig Diff**	Sig Diff
Stats	t-stat: 0.65-P val: 0.51	U Test: 36052.5-P val: 0.74	Stat: 89.56-Pval: 8.26

## 4 Discussion

The comparison of Macro derived versus GPT-4 Python script derived e-norms showed no significant statistical difference between the means of the Excel Macro data and the GPT-4 Python script data with the independent *t*-test and the Mann-Whitney *U* test in all 10 files used for comparison. The Levene’s test showed no significant statistical difference in the variances between the two methods in 5 out of 10 e-norms data, but a significant difference in the other 5.

This was to be expected from the outset however given that the plateau identification in the Excel e-norms Macro allows for manual and visual selection of the plateau region, leading to variability in the datapoints selected.

The GPT-4 Python script on the other hand uses a consistent algorithm to identify the plateau resulting in the selection of a different set of data points, making it robust and more easily reproducible.

As a medical discipline, clinical neurophysiology generates a variety of data types consisting of nerve conduction and muscle action potentials measures, waveforms of nerve conduction and motor unit action potentials, and EEG time series data and waveforms representing patterns of brain wave activity, among many others.

For research purposes, these data frequently require statistical and programming skills to help in their analysis.

But people with these skills live in different silos(Chatterjee, 2019) than clinical neurophysiologists do, requiring those to frequently reach outside their field to identify people with these skills, select the right ones for their project, and engage in communication, work, and data analysis with them, activities sometimes requiring months to put together, not to mention the necessary budgets to assemble.

As powerful as LLMs such as OpenAI’s GPT-4 have become, they occasionally create the impression that current models involving the recruitment of programmers and statisticians for research purposes may no longer be needed if such analysis can be done with GPT-4 alone.

This author’s experience has shown that such assumption is only partially correct because it uses the wrong heuristic in its distinction by simplifying the issue to programmers and statisticians versus AI, an issue that is at odds with the fundamental difference in the approach each uses.

While a human programmer and an AI like GPT-4 can write programs and perform statistical analysis by following the same principles, there are fundamental differences in how each approaches these tasks.

Human programmers typically engage in first attempting to understand the problem, clarifying potential ambiguities while identifying needs and limitations in the data being used. They are also adept at recognizing changes in the project and anticipating potential mistakes they've previously come across.

GPT-4 on the other hand typically operates based on data patterns, both good and bad, it encounters to develop solutions based on what it has been exposed to and trained on, all while lacking a programmer’s personal experience with the project at hand.

These differences can easily be experienced when one submits the same GPT-4 query at different times of day or days of the week and receives different answers from GPT-4 to the same query.

Such response “patterns” can be immediately recognized when working with a human programmer and serve to highlight the fundamental differences in how human programmers and GPT-4 approach the same task.

In fact, during this work, this author discussed how these approaches differ with GPT-4 asking for suggestions on how to mitigate these issues, and received the recommendations below. For economy of space, these suggestions were truncated but weren’t otherwise edited:

• “**Be Specific**: Clearly define what you are trying to achieve… The more details you provide, the better ChatGPT can tailor its responses to fit your needs”.
• “**Provide Context**: Explain where you are in your project and what technologies or tools you are using”.
• “**Outline What You’ve Tried**: Sharing what you've already attempted helps avoid suggestions you've explored and can help pinpoint where you might be going wrong if you're encountering issues”.
• “**Include Error Messages**: If you're dealing with bugs or errors, include the **exact** error messages you're seeing”.
• “**Break Down Large Problems**: If you're dealing with a complex issue, break it down into smaller, manageable parts and ask about each part separately”.

These are important facts to be aware of when using AI since over the past 3–4 years, there has been more and more clinical neurophysiology research projects developed using it in the fields of Electroencephalography (EEG), and Electromyography (EMG)(Ray et al., 2022), both of which explored the development of new algorithms that include the automation of procedures, improvement of diagnostic outcomes through the use of machine learning, and the unmasking of hidden insights in diagnostic rules to avoid the misinterpretation of studies due to signal noise, technical errors, and artifacts (Boyer et al., 2023).

Similar developments have taken place in fields such as the interpretation of X-Rays and mammograms (Oren et al., 2020, Leibig et al., 2022) that have greatly helped devise ways to allow for the earlier detection of abnormalities that may remain elusive with current human experts analysis methods.

In EEG in particular, an AI program called Standardized Computer-based Organized Reporting of EEG–Artificial Intelligence, or SCORE-AI (Tveit et al., 2023), has been recently developed to distinguish between normal and abnormal EEG studies and classify abnormalities into different diagnostic categories to facilitate clinical decision making.

SCORE-AI was developed using deep learning, a subset of machine learning that uses artificial neural networks to mimic the human brain’s learning process, and was trained on a total of 30,493 EEG studies and validated on 3 independent studies datasets of 9,785 EEGs (not used in its training) that were evaluated by 14 experts. On clinical testing, the program achieved high accuracy and performed at levels of accuracy similar to those of human experts.

To date, most AI systems used in EMG have consisted of studies designed for the identification and classification of resting membrane potential waveforms that include fibrillations and sharp waves and other resting membrane potentials (Nam et al., 2019, Gao et al., 2022). Other studies have used AI based classification of motor unit potentials in needle EMG recordings (Hubers et al., 2023, Yousefi and Hamilton-Wright, 2014).

In this work, the author chose to tackle an important downside in the interpretation of nerve conduction studies, one that relates to the availability of the appropriate normal values used in their interpretation.

Given the difficulty, time, and resources required to build one’s own normal values, EMGers tend to use normal values collected by others that would likely skew the interpretation of the studies they perform on their cohorts.

While this problem can be managed in adult cohorts where there is an abundance of published normal values in the literature, it is nearly impossible to manage in pediatric and geriatric cohorts where there is precious published few.

Using GPT-4 to automate the e-norms plateau identification with an algorithm to automatically and robustly derive normal values will greatly help to standardize this task.

During this work, it was worth noting that our experience with AI has revealed an important point that is worth mentioning. Given the occasional encounter of different answers to the same GPT-4 input depending on the day and time used, one can safely say that GPT-4 has not yet passed the Turing test (Turing, 1950) because we were easily able to tell that a human would have been unlikely to make the same mistake.

But throughout the work on this project, it has also become abundantly clear to the author that AI will greatly improve the ability of researchers not trained in programming or statistical analysis to travel much farther with an idea they wish to explore before needing to engage specialists in these fields, leading them to do so only when they have explored the validity, worthiness, and applicability of their research ideas while developing the right questions to explore with the experts.

What’s next for AI? According to a recent Nature Biomedical Engineering editorial, the next peer reviewer maybe an AI chatbot (The advent of human-assisted peer review by AI, 2024), a new reality we all must learn to adapt to and learn from.

## 5 Conclusions

In this work the author describes his experience using OpenAI’s ChatGPT-4 to automate the identification of an e-norms plateau for the development of nerve conduction studies normal values derived from a mixed dataset of patient studies containing normal and abnormal data.

The author highlights the benefits AI would bring to the analysis of clinical neurophysiology data, but also describes some of the limitations one needs to be aware of as this technology is still trying to learn from us and would benefit from our feedback, both good and bad.

Quoting Frank Uhlmann, a biochemist at the Francis Crick Institute in London “(AI) is just revolutionary… It’s going to democratize structural-biology research” (Callaway, 2024).

## Declaration of competing interest

The author declares that he has no known competing financial interests or personal relationships that could have appeared to influence the work reported in this paper.
